# Development of a Flexible MEMS Sensor for Subsonic Flow

**DOI:** 10.3390/mi13081299

**Published:** 2022-08-12

**Authors:** Koichi Murakami, Daiki Shiraishi, Shunsuke Mizumi, Yoshiko Oya, Naoto Omura, Takanori Shibata, Yoshiyasu Ichikawa, Masahiro Motosuke

**Affiliations:** 1 Department of Mechanical Engineering, Graduate School of Engineering, Tokyo University of Science, Tokyo 125-8585, Japan; 2 Research and Innovation Center, Mitsubishi Heavy Industries, Takasago City 676-8686, Japan; 3 Department of Systems Innovation Engineering, Faculty of Science and Engineering, Iwate University, Morioka 020-8551, Japan; 4 Department of Mechanical Engineering, Faculty of Engineering, Tokyo University of Science, Tokyo 125-8585, Japan; 5 Water Frontier Research Center, Research Institute for Science and Technology, Tokyo University of Science, Tokyo 162-8601, Japan

**Keywords:** MEMS flow sensor, hot-film, flow rate, flow direction, subsonic flow

## Abstract

Detection and control of flow separation is a key to improving the efficiency of fluid machinery. In this study, we developed a flexible MEMS (microelectromechanical systems) sensor for measuring the wall shear stress and flow angle in subsonic airflow. The developed sensor is made of a flexible polyimide film and a microheater surrounded by three temperature sensor pairs. The sensor measures the wall shear stress from the heater output and the flow angle from the temperature gradient around the heater. The geometry and design of the heater and temperature sensors were determined based on numerical simulations. To evaluate the validity of the sensor, we conducted an experiment to measure the wall shear stress and the flow angle in a wind tunnel in different velocities ranging from 30 m/s to 170 m/s, equivalent to Mach numbers from 0.1 to 0.5. The heater output was proportional to one-third power of the wall shear stress. Additionally, the bridge output correlating the temperature difference between two opposing temperature sensors showed sinusoidal variation depending on the flow angle. Consequently, we have clarified that the developed sensor can measure both the wall shear stress and flow direction in subsonic flow.

## 1. Introduction

The energy and transportation industries are the primary sources of greenhouse gas emissions that contribute to global warming [1]. These industries often use a variety of fluid machinery, such as gas turbines and aircraft engines, and there is a strong need to improve the efficiency of this fluid machinery to reduce the emission of greenhouse gas. One method to improve the efficiency is to suppress the boundary layer separation on the surface [2]. The flow separation tends to occur on curved surfaces, especially around complex three-dimensional parts. In such a case, separated flow can be very complex. Currently, computational fluid dynamics (CFD) and entropy analysis have often been used to optimize the blade geometry [3,4,5]. However, turbulence models such as RANS (Reynolds-averaged Navier–Stokes) cannot accurately predict the flow with separation. Therefore, there is still a high need for direct measurement of the near-wall flow to characterize and control the separated flow.

To detect the three-dimensional separation, it is important to measure the wall shear stress as well as the flow direction along the wall, especially on a curved surface. Although particle image velocimetry (PIV) [6] and fiber Bragg grating (FBG) [7] are used to detect the flow separation, these methods have drawbacks, including: (a) they require a large-scale measurement system, (b) detailed flow direction on the wall cannot be identified, (c) it is difficult to apply them in actual fluid machinery due to the need of optical access, and (d) it is difficult to simultaneously measure wall shear stress and flow direction. In this study, we utilized microelectromechanical system (MEMS) technology, which enables extremely compact and thin sensor devices and measurement in a small area with minimal influence on the flow [8] to develop the near-wall flow sensor, overcoming the above-mentioned drawbacks. Although many MEMS-based compact airflow sensors have been developed [8,9,10,11,12,13,14,15,16], they are fabricated on a solid Si substrate that is not suitable to be applied on a curved surface where flow separation frequently occurs. To expand the application for the curved wall, flexible flow sensors using polyimide or PET (polyethylene terephthalate) substrate have been developed [17,18,19,20,21,22,23,24,25]. However, they have no function to detect the flow angle. Although a sensor developed by Que et al. can detect the flow angle over a full range of 360° [26], the velocity range is limited to less than 30 m/s. Flexibility, capabilities of flow direction and angle, and maximum velocity of reported MEMS airflow sensors are summarized in Table 1. Although some sensors can measure flow direction and angle, most reported MEMS sensors have been developed to measure a low-velocity range of several m/s to several tens of m/s [9,10,11,12,13,14,15,16,17,18,19,20,21,22,23,24,25,26,27,28]. Therefore, it is necessary to investigate the potential of the development of a flexible flow sensor that can be applied for high-speed airflow.

The purpose of this study is to develop a flexible MEMS sensor enabling simultaneous measurement of wall shear stress and flow direction, and which can be installed on curved surfaces in fluid machinery where the flow is in the subsonic range faster than 150 m/s. In this article, the measurement principle of simultaneous measurement of wall shear stress and flow direction is described, with the sensor design based on numerical simulation and the fabrication process then presented. Experiments in a subsonic wind tunnel with a velocity range from 30 to 170 m/s (Mach numbers of 0.1–0.5) were conducted to confirm the validity of a prototype of the developed sensor. Sensor characteristics for the measurements of wall shear stress and flow direction were discussed based on the experimental results.

## 2. Measurement Principle

**Figure 1:**
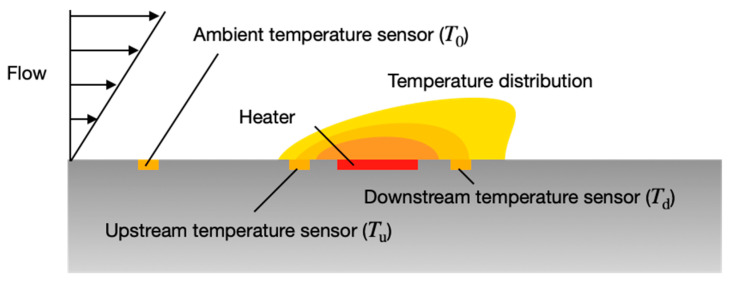
Principle of the thermal flow sensor for wall shear stress and flow angle.

In this study, the wall shear stress and flow angle were measured based on heat transfer around a heater made of a thin metal film on a flexible substrate because the thermal detection has been primarily used for airflow sensors [27]. Figure 1 shows a schematic of the thermal flow sensor in the present study. The sensor consists of a heater element, two temperature sensors near the heater, and a sensor for an ambient temperature. The sensor measures the wall shear stress from the heat balance of the heater and the flow angle from the temperature distribution around the heater.

The thermal energy of the heater is transferred by heat conduction to the wall and by the forced convection of the upper fluid. The correlation between the net heat *Q* transported by the fluid and the wall shear stress, *τ_w_*, which is widely used in commercial hot-film sensors, is used in our study. The net heat *Q* transported by the fluid and the wall shear stress *τ_w_* are expressed by Equation (1) [29,30].

(1) $$Q\propto \tau_{w}{}^{1/3}$$

since *Q* is strongly correlated with the power input required to maintain a constant temperature of the heater, *τ_w_* can be determined by measuring the heater power.

The flow around the sensor changes the temperature distribution induced by the heater, with the flow direction measured using the distribution. A different sensor for measuring the ambient temperature is located far away from the heater, with upstream and downstream temperature sensors placed near the heater to detect the temperatures upstream and downstream of it. A voltage is applied to the heater to maintain the temperature of the heater at a certain temperature, using a temperature *T*_0_ measured by the ambient temperature sensor. When there is no flow, an isotropic temperature distribution is generated around the heater due to heat conduction between the air and the substrate. In this case, both the upstream and downstream sensors show the same temperature (*T*_u_ = *T*_d_). In the presence of flow, the temperature distribution is distorted because of the forced convection, causing the decrease of the upstream temperature and the increase of the downstream temperature (*T*_u_ < *T*_d_). The difference between *T*_u_ and *T*_d_ provides information on the flow direction [8,31]. In the actual device, multiple pairs of temperature sensors were embedded around the heater (see Section 3.2) to achieve multidirectional measurement.

## 3. Design and Fabrication of the Sensor

### 3.1. Numerical Simulation for Sensor Design

The technical requirements for the sensor development in this study are: (a) the sensor is thin enough not to disturb the surrounding flow, (b) the sensor is flexible to be installed on a curved surface where the separation is likely to occur, (c) the sensor can measure flow angle and wall shear stress, and (d) the sensor should be applicable to high-speed subsonic flow. Here, we designed the flexible flow sensor to satisfy these requirements. In particular, numerical simulations were used to determine the size and position of the temperature sensors in the heater for flow angle measurement.

**Figure 2:**
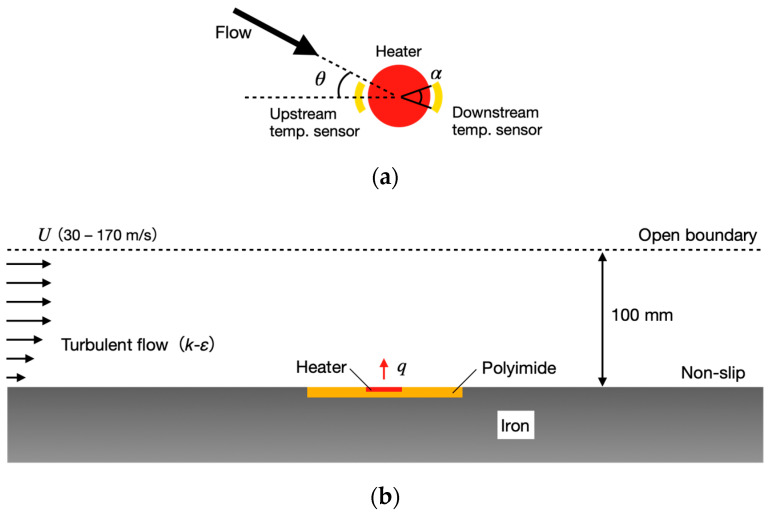
(**a**) Schematic of heater and temperature sensors for flow angle measurement with flow angle of *θ* and temperature sensor angle of α; (**b**) Numerical model for simulation. Heat energy generated at a microheater on a polyimide film embedded on the wall is transferred by fully developed turbulent flow with main flow velocity from 30 m/s to 170 m/s.

Figure 2 depicts a schematic diagram of the area around the sensor used in the simulation. The finite element method software COMSOL Multiphysics (v6.0, COMSOL Inc., Burlington, MA, USA) was used. A heater is located in the center, with several temperature sensors placed around it. The heater was circular in shape for axial symmetry, with 72 temperature sensors virtually placed at 5° increments to cover all the azimuth angles. The area of each temperature sensing was varied by obtaining the average temperature of several micro sensors (Figure 2a, where only two are shown in the case of sensor angle *α*). The temperature difference Δ*T* in a pair of two sensors located opposite each other with respect to the heater was used as the detected signal.

The continuity equation and compressible Navier–Stokes equation were used as the governing equations for the flow field, while the *k*-*ε* model was used as the turbulence model. For the thermal calculation, the energy equation was used. A constant heat flux was applied to the heater section, with the heat flux adjusted to obtain the average heater temperature of 50 K higher than the ambient temperature. The boundary conditions were an open boundary and a no-slip wall, as shown in Figure 2b, and mainstream velocities of 30 m/s to 170 m/s (equivalent to 0.1 to 0.5 in Mach numbers) were applied to investigate the temperature difference Δ*T* in difference flow conditions. Polyimide film with a thickness of 25 μm was used as the substrate and iron was used as the wall. The sensor was flush-mounted on the wall surface without any gap between them.

**Figure 3:**
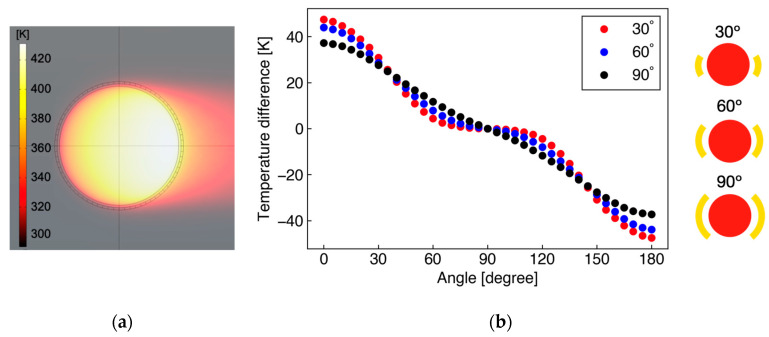
(**a**) Simulated temperature distribution around a heater; (**b**) Relationship between flow angle *θ* and temperature difference Δ*T* for different sensor angles α.

Figure 3a shows the simulation results of the temperature distribution around the heater with a diameter of 1000 μm. The main flow velocity is 100 m/s and small elements around the circular heater are temperature sensors located at 20 µm. The result shows that heat is mainly transported downstream and is hardly spread upstream, with there being almost no effect of heat conduction through the substrate. This fact implies that the distance between the heater and the temperature sensor can be relatively small. Therefore, in this study, the distance was set to 20 µm considering the fabrication accuracy. Figure 3b shows the temperature difference (Δ*T* = *T*_u_ − *T*_d_) between upstream and downstream sensors for the sensor angles α of 30°, 60°, and 90° (corresponding to sensor element numbers of 6, 12, and 18, respectively), as a function of the flow angle *θ*. In Figure 3b, Δ*T* shows the maximum at *θ* = 0°, zero at *θ* = 90°, and the minimum at *θ* = 180°. The angular characteristics from 0° to 90° are inversely symmetric with those from 90° to 180°. The sensitivity in the angle measurement can be evaluated from the slope of Figure 3b. In the small sensor angle α, the sensitivity is higher in small flow angle *θ*, whereas it worsens around *θ* ~ 90°. This sensitivity variation depending on *θ* is suppressed in large α. In this study, α = 60° was adopted for the actual sensor based on the simulation results, which exhibits intermediate characteristics. To measure multidirectional flow with the angle range of 360°, six temperature sensors (three pairs) were placed on the circumference with equal spacing of 5°, with the sensor angle α to be fabricated as 55°.

### 3.2. Sensor Fabrication

**Figure 4:**
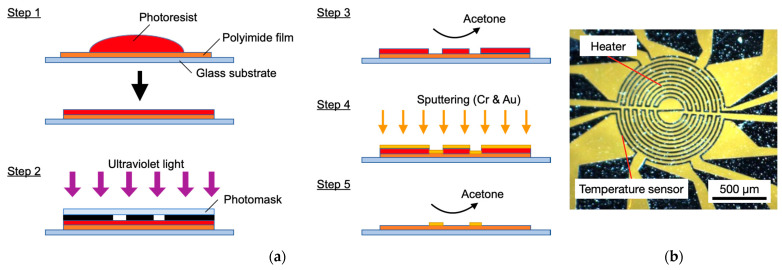
(**a**) Fabrication process of the flexible MEMS flow sensor; (**b**) Picture of heater and temperature sensor in the MEMS flow sensor. The yellow area is polyimide substrate and the black area is Au thin film.

Figure 4a illustrates the fabrication process of the sensor in this study. A 25 µm-thick polyimide film (Kapton^®^ 100H, Du Pont-Toray, Tokyo, Japan) with excellent heat resistance and mechanical durability was coated with a photoresist to create the patterns for the heater and temperature sensors (Step 1). Ultraviolet light was then irradiated through a photomask (Step 2) and the exposed area of the photoresist was removed using acetone (Step 3). Next, Cr for an adhesive layer and Au for the electrode were deposited by DC magnetron sputtering. The thickness of the Cr–Au electrodes was 10 nm and 100 nm, respectively (Step 4). The electrode shape was then fabricated by lift-off to remove unnecessary photoresist using acetone (Step 5). Figure 4b shows an image of the fabricated sensor. The heater diameter is 1000 µm, with a serpentine pattern used for the heater electrode to increase the resistance. The electrode widths of the heater and temperature sensors are 20 µm. A typical resistance of the heater and the temperature sensors were 400 Ω and 15 Ω, respectively. An ambient temperature sensor, not shown in Figure 4, was also fabricated on the same substrate. The temperatures of the center heater, surrounding temperature sensors, and ambient sensor were monitored by their resistances.

## 4. Experimental Results

### 4.1. Measurement System

**Figure 5:**
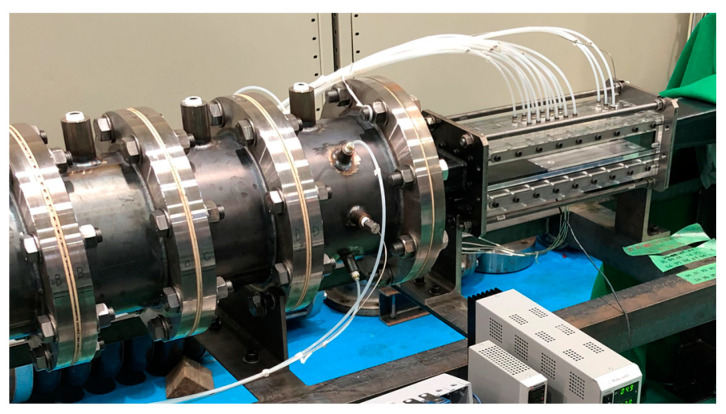
A picture of an experimental wind tunnel. The MEMS sensor is embedded on the surface wall of the test section in the fully developed high-speed subsonic flow.

As a proof-of-concept of the developed flow sensor, the wall shear stress and flow angle measurement in a high-speed subsonic flow in a wind tunnel were performed. Figure 5 shows a picture of the test section in the wind tunnel in the present study. The width and height of the test section are 10 mm and 100 mm, respectively. The sensor was flush-mounted on the wall 500 mm downstream from a nozzle to secure a fully developed turbulent flow in the test section. The relative angle between the senor and the main flow *θ* was controlled by rotating the sensor from the range of 0° to 190°. As a reference for the wall shear stress at the sensor location, a Preston tube with an inner diameter of 0.7 mm and an outer diameter of 1.0 mm was placed next to the sensor. The calibration curve between Mach number and wall shear stress was obtained from Bradshaw’s relation [32]. The flow angle and wall shear stress were measured in the range of Mach numbers M = 0.1–0.5 (approximately 30–170 m/s in bulk velocity). The heater temperature was kept constant by PID (proportional–integral–derivative) control [33] at 50 K higher than the ambient temperature. The resistance difference caused by the temperature difference between the two temperature sensors was measured by a Wheatstone bridge circuit every 0.5 s. The voltage to the heater was supplied from a benchtop DC power supply (P4K40-0.6, Matsusada Precision Inc., Shiga, Japan) via a printed circuit board, with the signals from the sensors recorded to a computer using LabVIEW (NI, Austin, TX, USA) via the same printed circuit board. A typical voltage and current applied to the heater were 6 V and 15 mA, respectively.

### 4.2. Wall Shear Stress Measurement

**Figure 6:**
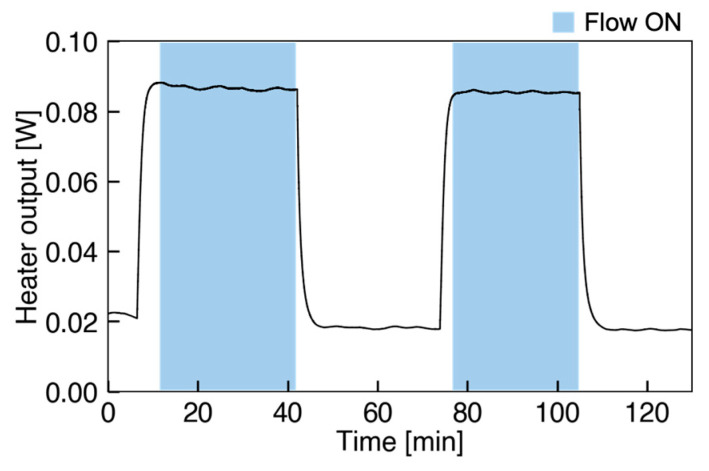
Heater output of changing flow condition.

Firstly, the time variation of the heater output (heater power) during constant flow rate was investigated. In this test, the sensor was first exposed to the constant flow for about 30 min, then the airflow was stopped for 30 min, and this was repeated twice. The time series of the heater output in this experiment is shown in Figure 6. The blue areas correspond to the period with the flow, while the other areas are in no-flow condition. From the result, it is clearly shown that the sensor outputs indicate constant values during the flow and that the outputs in the first and second flow show almost the same values. During the flow, the sensor output was very stable with a standard deviation of 0.5%. Moreover, the reproducibility and no hysteresis were confirmed in this experiment.

**Figure 7:**
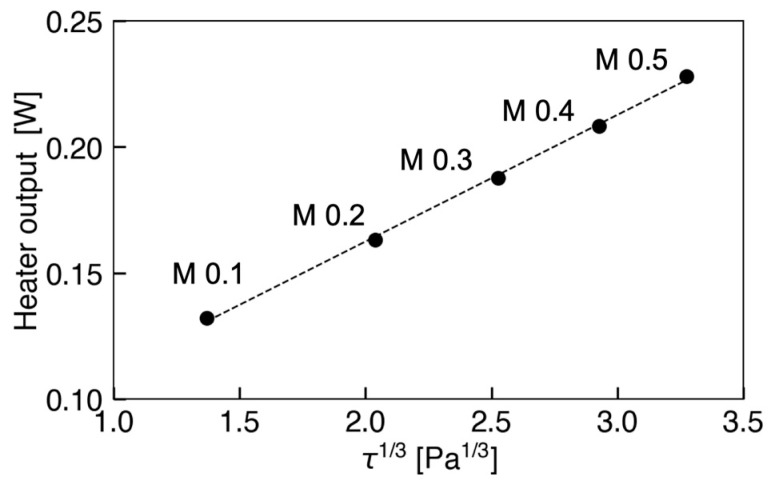
Relationship between heater output and *τ*^1/3^ at each Mach number.

Next, the relationship between the heater output from the sensor and 1/3 power of the wall shear stress, *τ_w_*^1/3^, in Mach numbers ranging from 0.1 to 0.5 is depicted in Figure 7. Here, the *τ_w_* shown was obtained from a preliminary measurement using the Preston tube. The result indicates that the heater output is proportional to *τ_w_*^1/3^ with a coefficient of determination of 0.9986. Consequently, we confirmed that the developed flow sensor is applicable to the measurement of wall shear stress up to approximately 35 Pa in the high-speed subsonic flow.

### 4.3. Flow Angle Measurement

**Figure 8:**
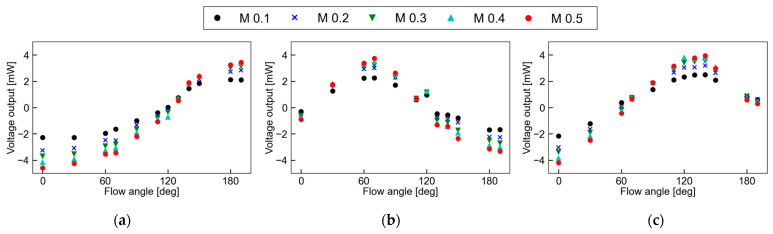
Relationship between flow angle and voltage output of each sensor: (**a**) sensor a; (**b**) sensor b; (**c**) sensor c.

Figure 8a–c shows the relationship between the flow angle and the voltage output in the bridge circuit from the sensor pair at M = 0.1–0.5. The sensor a-c represents each sensor pair, which are installed at angles of 0°, 60°, and 120° to the streamwise direction, respectively. From Figure 8a–c, it can be seen that the flow angle of each sensor pair varies sinusoidally depending on the Mach number, which is similar to previous results using multiple microheaters [26]. The maximum voltage outputs were observed at around 190° for sensor a, 70° for sensor b, and 140° for sensor c, respectively. The angle at which the voltage output reaches its extreme value is approximately the same as the installation angle. A deviation of 10° from the designed angle was observed; however, this was due to an angular error in the installation of the sensor. The difference between the maximum and minimum values, i.e., the amplitude, increases as the Mach number increases, suggesting that the sensitivity of angle detection increases as the velocity increases.

**Figure 9:**
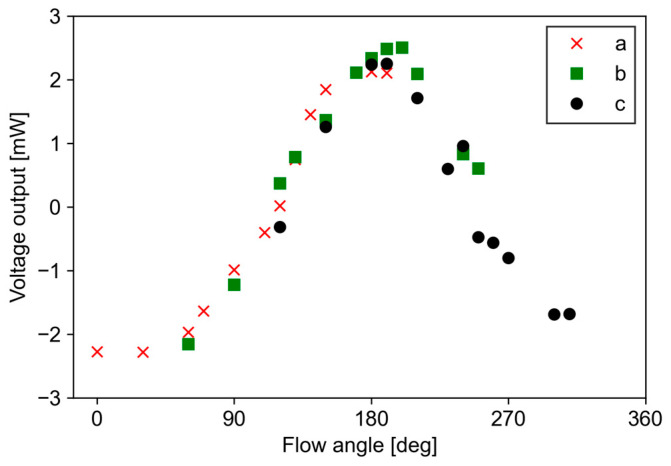
Flow angle dependence on voltage output from each temperature sensor pair. a, b, and c in the legend correspond to sensors a, b, and c, respectively.

Figure 9 shows superimposed outputs from the three sensor pairs at M = 0.3. The data used are the same as in Figure 8a–c. In this superposition, sensor b is shifted by 60° and sensor c is shifted by 120°, corresponding to the installation angle of the sensor pairs. This result shows that the voltage outputs of the three sensor pairs are on a single sinusoidal curve. Therefore, it becomes possible to calculate the flow angle using this characteristic. The similar trend of the single sinusoidal curve in each data was also confirmed in other Mach numbers.

**Figure 10:**
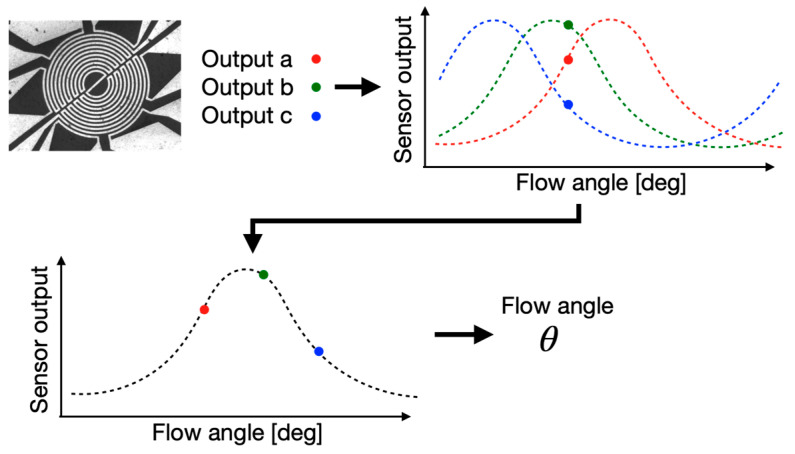
Determination process of flow angle by the developed sensor. Three sensor outputs are fitted to the calibration curve after shifting the installation angle for each sensor.

**Figure 11:**
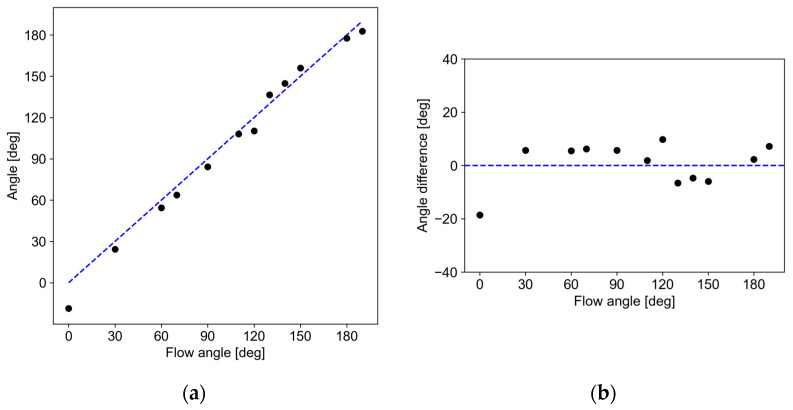
(**a**) Measured flow angle under high-speed subsonic flow; (**b**) Deviation of the measured flow angle.

The determination process of the flow angle *θ* from outputs from the three pairs of temperature sensors is schematically depicted in Figure 10. Since the three sensor outputs have the same dependence on the flow angle as in Figure 9, curve fitting was performed using experimentally obtained sensor outputs, taking the installation angle difference into account. Figure 11a shows the measurement results of the flow angle in the wind tunnel test in this study. The horizontal and vertical axis in the figure is the actual flow angle and measured angle by the developed sensor, respectively. Figure 11b shows the deviation of angle. The measurement results indicate that the measured angle has an error of less than 10° except for 0°. At 0°, the deviation was approximately 18°. This large error would be due to the defect in the fabrication of sensor a, which shows several ohms difference; this error can be reduced by optimizing the fabrication process including the electrode design, or by introducing an amplifier into the bridge circuit. Consequently, it can be stated that the developed flow sensor can measure the flow angle in a high-speed subsonic region.

## 5. Conclusions

In this study, a flexible multidirectional MEMS flow sensor using a polyimide substrate, which is applicable to high-speed subsonic flow, was developed. The sensor measures the wall shear stress by the thermal balance of convective heat transfer and the flow direction by the temperature distribution around the microheater. Three temperature sensor pairs enable multidirectional flow measurement. As a result, it was confirmed that the developed sensor is applicable in high-speed flow from 30 m/s to 170 m/s (Mach numbers from 0.1 to 0.5) without any damage to the sensor. The wall shear stress can be determined by the 1/3 power law of the heater input. The flow angle can be measured by the fitting based on the calibration of the three sensor outputs. The sensor can be utilized for curved walls to evaluate complicated separated flow, which often occurs in various fluid machinery.

## Figures and Tables

**Table 1:** Hard and soft MEMS-based airflow sensors and their capabilities (Y = yes, N = no).

Type	References	Flexibility	Direction	Angle	Max. Velocity [m/s]
Hard	9	N	N	N	45
	10	N	N	N	63
	11	N	N	N	45
	12	N	Y	N	73
	13	N	Y	N	19
	14	N	Y	N	5
	15	N	Y	Y	10
	16	N	Y	Y	1
Soft	17	Y	N	N	7.5
	18	Y	N	N	25
	19	Y	N	N	1.5
	20	Y	Y	N	15
	21	Y	N	N	25
	22	Y	N	Y	14
	23	Y	Y	N	33
	24	Y	N	Y	13
	25	Y	Y	N	1.5
	26	Y	Y	Y	30
	This study	Y	Y	Y	170

## Data Availability

The data presented in this study are available from the corresponding author upon request.
